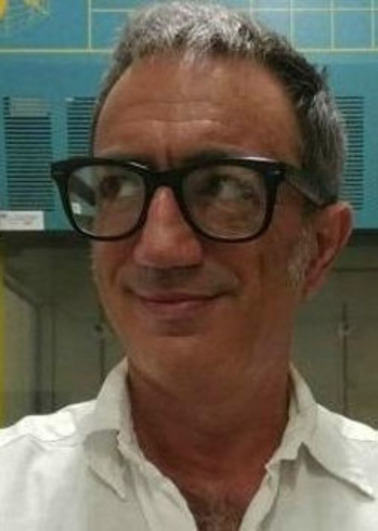# Harvesting Light To Produce Heat: Photothermal Nanoparticles for Technological Applications and Biomedical Devices

**DOI:** 10.1002/chem.202102123

**Published:** 2021-09-30

**Authors:** Piersandro Pallavicini, Giuseppe Chirico, Angelo Taglietti

**Affiliations:** ^1^ Department of Chemistry Università degli Studi di Pavia v. Taramelli 12 27100 Pavia Italy; ^2^ Department of Physics “G. Occhialini” Università Milano Bicocca p.zza della Scienza 3 XX100 Milano Italy

**Keywords:** antibacterial, anticounterfeit, nanofluids, nanoparticles, photothermal effect

## Abstract

The photothermal properties of nanoparticles (NPs), that is, their ability to convert absorbed light into heat, have been studied since the end of the last century, mainly on gold NPs. In the new millennium, these studies have developed into a burst of research dedicated to the photothermal ablation of tumors. However, beside this strictly medical theme, research has also flourished in the connected areas of photothermal antibacterial surface coatings, gels and polymers, of photothermal surfaces for cell stimulation, as well as in purely technological areas that do not involve medical biotechnology. These include the direct conversion of solar light into heat, a more efficient sun‐powered generation of steam and the use of inkjet‐printed patterns of photothermal NPs for anticounterfeit printing based on temperature reading, to cite but a few. After an analysis of the photothermal effect (PTE) and its mechanism, this minireview briefly considers the antitumor‐therapy theme and takes an in‐depth look at all the other technological and biomedical applications of the PTE, paying particular attention to photothermal materials whose NPs have joined those based on Au.

## Graphical abstract

**The photothermal effect** is the ability to transform absorbed electromagnetic radiation into heat. Although it was first studied in nonspherical Au nanoparticles with NIR localized surface plasmon resonance absorptions for through‐tissues hyperthermal tumor ablation, for more than 20 years this effect has been recognized in nanoparticles of different materials and shapes, and used for a wide spectrum of biomedical and technical applications, which are reviewed here.

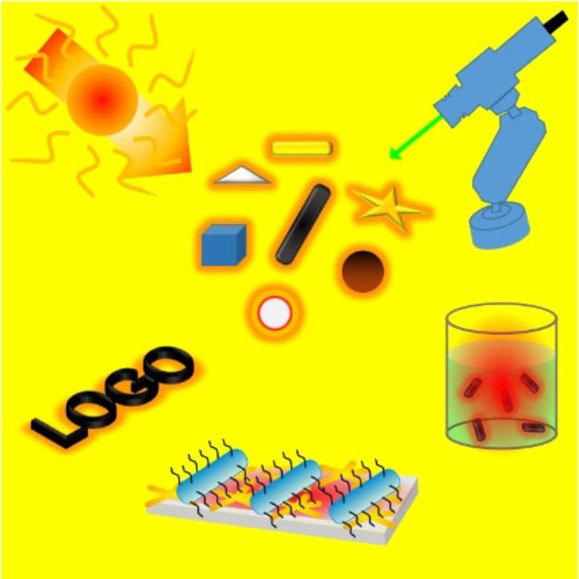

## 1 Introduction

The phenomenon of the conversion of light into heat is named the “photothermal effect” (PTE). Generally speaking, it consists of preferred thermal versus emissive relaxation of any quantum system that absorbs light. NP absorb much more intensely than molecules at specific resonances. This leads to the intuitive idea of using NP with well shaped absorption bands, characterized by a *λ*_max_, for example, like those due to localized surface plasmon resonance (LSPR) in noble metal NP, and irradiating devices capable of hitting as precisely as possible such *λ*_max_, like laser sources. While this is indeed the most frequent case, also nanoparticles with large, poorly defined but intense absorptions (like copper sulfide NP or carbon nanotubes) present a huge PTE when irradiated at any wavelength in their absorbing range. On the other hand, continuum wide‐range radiation sources, like diode arrays or sunlight, can also be used on NP to convert light into heat.

AuNP and AgNP, under laser irradiation, were the first systems in which the PTE was studied. The earliest papers, dating back to the 1980 s^[1]^ and 1990s,[^2^, ^3^, ^4^, ^5^, ^6^, ^7^] regarded the mechanism and kinetics of relaxation after light absorption from high energy pulsed lasers. Such studies evidenced the fast (femtoseconds) excitation of the conductive band electrons in spherical Au and Ag NP, followed by the electron cloud thermalization and the electron–phonon and phonon–phonon relaxation, leading to NP heating in a relative longer time (hundreds of picoseconds). In this kind of studies high energy pulsed lasers were used and shape change or even melting of Au nanospheres^[8]^ or nanorods^[9]^ was observed. Reviews on this topic and approach appeared since 1997^[10]^ forming as early as 2000 an exhaustive picture of the phenomenon.^[11]^

The idea of studying and exploiting the PTE of NP for a biomedical application first came in the early 1990s, when it was found that carbon NP heated with a pulsed laser produced cavitation and acoustic waves in an aqueous medium.[^12^, ^13^] The same phenomenon studied on a pigment (melanin) nano‐ and microparticles lead to the concept that cells containing NP can be selectively killed by local heat generation.[^14^, ^15^, ^16^] However, these studies still employed high energy pulsed lasers sources in the visible range (typically at 536 nm), that is, at a wavelength at which skin, blood and muscles are not transparent and may obviously be damaged. A turning point was the attainment of aqueous solutions of Au nanorods (especially when straightforward wet seed‐growth syntheses were discovered in 2001[^17^, ^18^, ^19^]) as well as of silica‐core gold nanoshells, that were first prepared in 1998.^[20]^ As both Au nanorods and nanoshells have intense LSPR adsorptions that can be tuned in the Near IR (NIR), and particularly inside the first biotransparent window (700–950 nm^[21]^) where water, blood, skin and muscles absorb weakly, a burst of publications on photothermal through‐tissues antitumoral therapies took place between 2005 and 2006.[^22^, ^23^, ^24^, ^25^, ^26^, ^27^, ^28^, ^29^, ^30^, ^31^] Literature never stopped to grow on this topic, with more NIR‐absorbing AuNP added to the arsenal, such as nanocages,^[32]^ clusters of nanospheres,^[33]^ nanostars,^[34]^ and the treatment of tumors with AuNP has become the literature area in which the PTE is more frequently exploited. The topic has been already extensively reviewed[^35^, ^36^, ^37^, ^38^] and will be only briefly mentioned here. This minireview is instead focused on the different uses of photothermal NP that have emerged in the new millennium. On the biological and medical side, photothermal NP are now used for cell stimulation, antibacterial and antibiofilm coatings, and for sterilizing medical devices such as flexible films for wound disinfection and healing. Purely technological applications of the PTE of NP have also attracted a lot of attention, in particular for solar heaters and solar steam generators, and for anticounterfeit thermally encrypted printing with nano‐inks. Moreover, this review takes into account the wide arsenal of photothermal NP that has recently joined AuNP. As absorption in the NIR remains in most cases a sought after feature, this has lead to the use of NIR‐absorbing and photothermally relaxing NP of materials such as Prussian blue,[^39^, ^40^] polypyrrole,[^41^, ^42^] porous silicon,[^43^, ^44^] carbon (graphene,[^45^, ^46^] fullerene,[^47^, ^48^] nanotubes,[^49^, ^50^, ^51^] nanohorns[^52^, ^53^]), copper sulfide,[^54^, ^55^, ^56^] silver,[^2^, ^7^, ^57^, ^58^] iron oxides,[^59^, ^60^] tungsten oxides,[^61^, ^62^] molybdenum chalcogenides,[^59^, ^63^, ^64^] titanium oxide,[^65^, ^66^] p‐block metals chalcogenides[^67^, ^68^] black phosphorus[^69^, ^70^, ^71^] and NIR‐absorbing molecular dyes.^[72]^ In the last case, a supramolecular approach is often designed, in which indocyanine green (IDG),[^73^, ^74^, ^75^] zinc phthalocyanine[^76^, ^77^] or multiple dyes[^78^, ^79^, ^80^] are included in NP of non absorbing materials (e. g., silica, organic polymers), this preserving their photostability but promoting quenching or self‐quenching processes that increase the thermal vs emissive relaxation.

## 2 PTE: Basic Concepts and Mechanisms

Photothermal NP are obtained with a huge range of materials and different mechanisms drive the conversion of light into heat. The most studied case is that of noble metals.^[81]^ Au, Ag, Cu and Pt NP have intense LSPR bands. As it has been already pointed out in the introduction, when these NP are photoexcited in the range of their LSPR bands, the plasmon oscillations relax nonradiatively by electron–electron and electron–lattice phonon collisions. This fast process (<1 ps) is followed by a relatively slower (∼100 ps) phonon‐phonon relaxation, leading to nanoparticle heating and heat transfer to the surrounding medium.[^2^, ^3^, ^4^, ^5^, ^6^, ^7^, ^8^, ^9^, ^10^, ^11^] The same light to heat conversion mechanism holds in different nanomaterials featuring LSPRs. This is the case of semiconductor nanocrystals with appreciable free carriers concentration, like Cu chalcogenides,[^82^, ^83^, ^84^, ^85^] zinc oxide,^[86]^ ITO (indium tin oxide),^[87]^ nonstoichiometric titanium oxides^[88]^ or molybdenum^[89]^ oxides. In these NP the LSPR band maximum can be tuned (usually in the NIR region) by tuning the doping degree and/or the stoichiometry. WO_3‐δ_ nanorods with *λ*_max_=900 nm are obtained when *δ*=0.17.^[61]^ Cu_2_Se (all Cu in oxidation state I) has no NIR absorption, but controlled, partial oxidation to Cu_2‐x_Se gives origin to an LSPR absorption band with *λ*_max_ ranging from ∼1500 to ∼1000 nm, in excellent correspondence with the bands calculated using the electrostatic approximation for the interaction of a conductive nanosphere with an external electric field and *x* ranging from 0.1 to 0.2.[^56^, ^82^]] NP made of polymers,^[90]^ like polypyrrole[^41^, ^42^] and polyaniline[^91^, ^92^, ^93^] exploit the vibrational relaxation of the electronic transitions in the conjugate polymers, that are non luminescent materials. In the case of indocyanine green (ICG)[^73^, ^74^, ^75^] the low fluorescence quantum yield of this molecule (3.9 % in water^[94]^) favors thermal relaxation. Inclusion of multiple molecules in a NP may change the ICG absorption spectrum^[74]^ but does not influence its photothermal behavior, while greatly enhances ICG photostability and solubility.^[94]^

**Figure 1:**
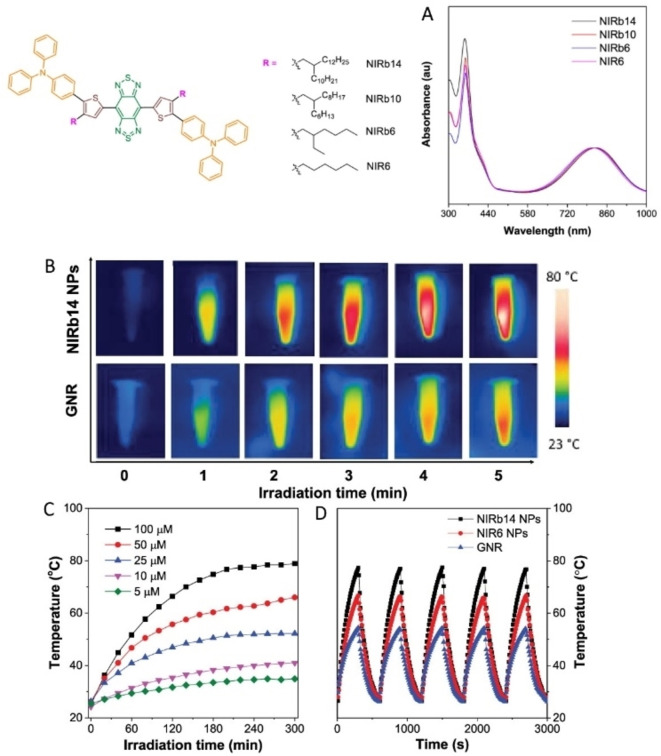
Formula of the molecules used in ref. [96] featuring molecular rotors and bulky alkyl chains grafted to the central donor–acceptor thiophene–thiadiazole moiety. A) Their normalized absorption spectra in THF. B) Thermal images of the NIRb14 molecule segregated in polymer NP, compared with Au nanorods (GNRs), under 808 nm laser irradiation (0.8 W/cm^2^) for different times. C) Thermograms (*T* vs time) of NIRb14 polymer NP at different concentrations (5–100 μM), *λ*_irr_=808 nm laser irradiation. D) *T* vs time in repeated irradiation/cooling cycles, for NIRb14 and NIR6 N, compared with GNRs. Adapted and reproduced with permission from ref. [96]. Copyright: 2019, American Chemical Society.

Aggregation of molecular fluorophores into NP, or in the core of NP of a different, nonabsorbing material, is an approach recently developed to obtain a fluorescence boost, a phenomenon defined by the acronym AIE, aggregation‐induced emission.^[95]^ However also the competitive thermal relaxation can be promoted in these systems, and the balance between fluorescence emission and thermal conversion can be tuned. In particular, rigidification of the local environment favors emission, while flexibilization drives to a twisted intramolecular charge transfer, that enhances the photothermal response.^[95]^ In this regard, molecular rotors and bulky alkyl chains grafted to a central donor‐acceptor thiophene‐thiadiazole moiety give strongly absorbing (at ∼800 nm) and highly photothermal multicomponent molecular aggregates, once segregated as NP into a poly(β‐amino ester)‐b‐poly(caprolactone) polymer shell^[96]^ (Figure 1). Such NP have a thermal conversion superior to that of Au nanorods, and display excellent stability in repeated irradiation/cooling cycles (Figure 1B–D). Similarly, conjugated tetraphenylethylene and naphthalene diimide‐fused 2‐(1,3‐dithiol‐2‐ylidene)acetonitriles have high molar absorptivity and give strong twisted intramolecular charge transfer in pegylated polymer NP, resulting in a huge PTE.^[97]^ In this context, the combination of AIE by luminogen‐functionalized mesoporous silica NP with the thermal action of conjugated NP displaying PTE, is a recently introduced approach that allows to obtain nanotheranostic systems for imaging‐guided chemo‐ and photothermal synergistic therapy for cancer.^[98]^ Single‐walled carbon nanotubes (SWCNT) has a semiconducting versus metallic character that depends on their diameter and chiral wrapping angle, resulting in multiple sharp absorptions between 450 and 1600 nm, due to interband transitions associated with van Hove singularities.^[99]^ Individual, separated SWCNT fluoresce intensely, but when associated in bundles vibrational relaxation is preferred,^[99]^ with strong enhancement of the photothermal yield.[^49^, ^50^, ^51^, ^100^] Prussian blue (PB) is a coordination polymer alternating [Fe(CN)_6_]^4−^ centers (Fe in oxidation state II) with Fe^3+^ ions octahedrally coordinated by the N atoms of the cyanide ligands.^[40]^ PB NP absorb at ∼700 nm thanks to a charge transfer electronic transition from Fe^II^ to Fe^III^ centers,^[101]^ followed by non‐emissive relaxation.

**Figure 2:**
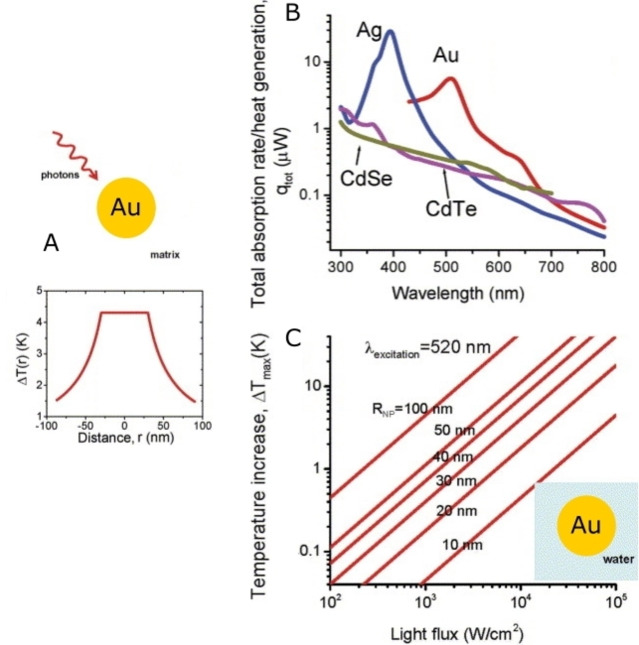
A) calculated *T* increase for a single AuNP (*r*=30 nm) as a function of the distance from the NP center (surrounding medium=water, irradiance 10^4^ W/cm^2^). B) Calculated rate of light energy dissipation in Au, Ag, CdTe, and CdSe NP, as a function of *λ*_irr_ (NP *r*=30 nm, irradiance 5×10^4^ W/cm^2^, surrounding medium=water). C) calculated *T* increase at the surface of single AuNPs of different radius in water, as a function of light flux (i. e., irradiance), when irradiating at 520 nm (LSPR band). Adapted from ref. [101] with permission. Copyright: 2007, Elsevier.

The PTE is typically observed for NP in colloidal solutions or inside a macroscopic solid matrix and the heat transfer from the NP to the solvent/matrix must be considered. The *T* increase for a single spherical AuNP has been modeled, and it is directly proportional to the intensity of the incident light and to the square of the NP radius (for spherical NP).^[102]^ Given the *T* of the NP, the Δ*T* of the surrounding medium decreases proportionally to *V*_NP_/*rk*_0_, with where *r* is the distance from the center of the NP, *k*_0_ is the thermal conductivity of the surrounding medium, and *V*NP is the NP volume (Figure 2A).The heat generation rate and the temperature increase depend on the material and in particular on the imaginary part of its dielectric constant.^[102]^ Figure 2B compares the calculated heat generation for spherical NP (*r*=30 nm) of different materials (Au, Ag, CdSe, CdTe) as a function of the irradiation wavelength. The profiles are coherent with the intuitive notion that the maximum heat is obtained when irradiating at the LSPR maximum, as it has also been experimentally demonstrated elsewhere.^[103]^ The lower heat generation of CdSe and CdTe is attributed to the different heating mechanisms, assigned to interband transitions in stoichiometric semiconductors.^[102]^ Interestingly, when the *T* increase on the surface of spherical AuNP of different radii is calculated as a function of the irradiance (W/cm^2^), Figure 2C is obtained. For small spherical AuNP (*r*=10 nm) the *T* increase of a single NP is ≪1 K unless a very high light flux (>10^4^ W/cm^2^) is used.^[104]^ Laser irradiances ≪10 W/cm^2^ (at 800 nm) are commonly used for technological and medical applications, also because the American National Standard Institute has established a maximum laser irradiance of 0.33 W/cm^2^ for exposure of skin.^[105]^) This obviously indicates that the commonly observed 5–50 K temperature increases are due to collective effects.^[106]^ A method to define the efficiency of transducing incident light to heat by suspensions of NP was defined for Au^[107]^ and is now used for all materials. Reported values range from 9.9 % (20 nm Au nanospheres^[107]^) to 13 % (Au nanoshells),^[108]^ 21 % (Au nanorods),^[108]^ 22 % (Cu_2‐x_Se),^[108]^ 28 % (black phosphorus NP),^[69]^ 55 % (TiO_2_ core‐shell NP).^[66]^ It has to be remembered that most of these values are obtained irradiating at 808 nm, that is the wavelength of the commonly available NIR lab bench laser sources (a wavelength well placed inside the biotransparent window^[21]^) but a good match between the maximum of absorption of a given NP and the irradiation wavelength may obviously optimize the photothermal conversion efficiency. In turn, the wavelength of the absorption maximum of a NP is tuned not only by its composition, but also by its dimensions and shape.^[11]^

## 3 Biomedical Applications of PTE Using NPs

### 3.1 Antitumoral photothermal therapy

Since the first pioneering papers of 2005–2006[^22^, ^23^, ^24^, ^25^, ^26^, ^27^, ^28^, ^29^, ^30^, ^31^]] thousands of articles have been devoted to the photothermal treatments of tumors by means of nanoparticles. The area has been extensively reviewed, often including papers presenting the theranostic use of functionalized photothermal NP: fluorophores conjugated to NP may allow imaging, the *T* increase may promote the release of drugs, photoacoustic diagnostics may be associated to the photothermal therapy.[^109^, ^110^, ^111^] The interested reader is addressed to the many reviews that are focused on NP of a given material like Au,[^35^, ^112^, ^113^, ^114^] C,[^47^, ^115^, ^116^] Si,^[44]^ P,^[117]^ Prussian blue,[^40^, ^118^] or adopt a generalist approach.[^59^, ^119^, ^120^] The general scheme is to use NP presenting PTE, stabilize them with a coating that also imparts a stealth effect (in most cases poly(ethylene glycol), PEG), and often adding a targeting ligand on the NP surface, selective for the tumoral cells line to be treated. In‐vitro or in‐vivo studies (on murine models) are carried out with laser treatments, in which the irradiating wavelength of choice is 808 nm. The local *T* reached in threated tissues should remain in the clinically relevant temperature range (41–48 °C).^[121]^ Moreover, if clinical through‐skin tissue treatments are envisaged, at least in perspective, the irradiance of a continuous laser source should be lower than the ANSI imposed limits of 0.33 W/cm^2^.^[105]^ In this regard, while the local *T* is often evaluated, especially in in‐vitro experiments (using a thermocouple or a thermal camera), the laser irradiance frequently largely exceeds the ANSI limits, or it is simply not given, as many papers report only the laser power and not the beam waist (i. e., the area impinging on the treated sample), this leaving undefined the possibility of using a given treatment in a clinical protocol. To bypass these weak points, different strategies may be pursued, such as fine tuning the wavelength of NP absorption bands so to have the best match with the available laser sources,^[108]^ or alternatively using laser sources at wavelengths longer than 808 nm,[^95^, ^98^] as well as increasing the efficiency of light to heat conversion^[66]^ and increasing, in the tumor site, the concentration of NP displaying PTE.

### 3.2 Cells stimulation

Self‐assembled monolayers (SAM) of NP have an established effect on controlling 2D cell cultures,^[122]^ and laser‐induced local heat generation stimulates the neural cells activity and proliferation, as it was observed that NIR laser pulses absorbed by water produce a local *T* increase that reversibly alters the electrical capacitance of the neurons plasma membrane, producing transient currents.^[123]^ These two observations were recently put together in studies that exploit NP to photothermally stimulate and control neural cells growth and differentiation^[124]^ Irradiation with NIR light has a positive stimulatory effect on the neurite length of neuronal cells cultured with gold nanorods^[125]^ and internalized Au nanorods irradiated on their LSPR band at 780 nm induce intracellular calcium transients in neuronal cells.^[126]^ Remote activation of neural tissues was demonstrated using the PTE of high aspect ratio Au nanorods with LSPR maximum at 980 nm.^[127]^ Using a laser at such long and low energy wavelength, the thermal activation of the plasma membrane in axons triggered action potentials of in vivo neural tissues, with an approach suitable to remotely manipulate neural systems and treat neurological disorders.^[127]^ NIR pulsed laser (780 nm) was used also to stimulate cultured rat primary auditory neurons after incubation with SiO_2_‐coated Au nanorods, obtaining 0.5–6 °C *T* increases (depending on the pulse duration) that correlated well with the observed electrical activity of the neurons on exposure to the laser pulses.^[128]^ On the other hand, exposure to continuous 780 nm laser for longer times (1–30 min) and with larger Δ*T* (10 °C) lead to inhibition of the neural activity in networks of primary cultured hippocampal neurons bearing Au nanorods bound to the plasma membrane,^[129]^ a result that is in agreement with the known inhibitory effect on neural activity exerted by sustained environmental *T* increase. Recently, a liquid crystal graphene oxide‐Au nanorod nanocomposite was prepared with LSPR absorption at 798 nm. Deposition of this composite on electrodes generates surfaces that support adhesion, proliferation, and differentiation of neuronal cells, in an ideal device for simultaneous electrical an NIR photothermal cell stimulation^[130]^ Carbon nanomaterials have been proposed for promote nerve regenerations:^[131]^ in this regard, it is interesting to report that pulsed laser irradiation (at 532 nm) of graphene layers favors the differentiation of human neural stem cells into neurons, although the thermal aspects have not been considered in this research.[^132^, ^133^]

Intracellular delivery of exogenous macromolecules into living cells has also took advantage of the PTE, with approaches resembling membrane‐disruption techniques. AuNP were made to aggregate on the surface of planktonic cells, obtaining membrane poration by irradiation with laser and consequent local *T* increase,[^134^, ^135^] this promoting macromolecular trafficking across cell membranes. A recent, smart evolution of this concept lead to a firmly grafted AuNP layer on silicon slides, obtained by plating. On 30–90 s irradiation with NIR laser (808 nm, irradiance in the 2.3–5.1 W/cm^2^ range) the local *T* increase lead to enhanced membrane permeability of surface‐adhering mouse embryonic fibroblasts and human umbilical vein endothelial cells. This in turn switched the efficient diffusion of macromolecules (dextran and plasmid DNA) from the surrounding medium into the cells cytosol. The AuNP have no adverse effect, also because they do not detach from the Si surface.^[136]^ A further development of this research lead to polydopamine/poly(*N*‐isopropylacrylamide) hybrid films. Polydopamine has the role of generating a local *T* increase by PTE, promoting cells poration and through‐membrane trafficking, while the thermoresponsive poly(*N*‐isopropylacrylamide) component allows the facile detachment and collection of the transfected cells.^[137]^

### 3.3 Monolayers of photothermal nanoparticles on bulk surfaces: switchable disinfecting materials

The use of the PTE of NP for disinfection is imagined mainly for surfaces of common use (e. g., public touch screens or hospital handles) to avoid infection transmission by contact, and as coatings for indwelling medical devices (e. g., prostheses or catheters) to avoid the formation or to remove bacterial biofilms. Monolayers of noble metal NP are grafted on bulk surfaces using a modification of the layer‐by‐layer technique.^[138]^ A first molecular monolayer is covalently grafted on the chosen surface, and it must possess outward functions allowing specific interactions with chosen noble metal NP.^[139]^ The NP monolayer is obtained with a second self‐assembling step, on the functionalized surface.

**Figure 3:**
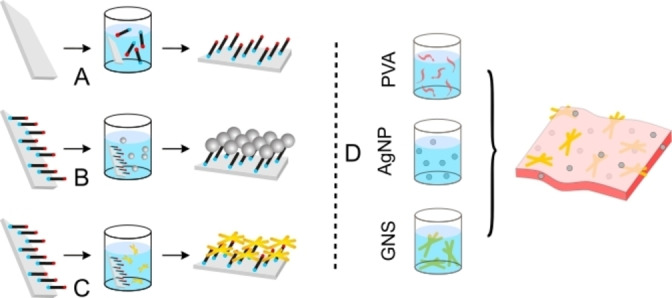
A)–C) Sketch of the layer‐by‐layer process leading to a monolayer of noble‐metal nanoparticles. Adapted with permission from ref. [134]. Copyright: 2018, Wiley‐VCH. D) Sketch of the one‐pot process to obtain PVA films containing both AgNP and gold nanostars. Reproduced from ref. [163], an open access MDPI 2021 publication.

This is sketched in Figure 3A (1st step) and 3B, C (2nd step) for the common case of a wet approach that allows to obtain AgNP^[140]^ or Au nanostars^[141]^ monolayers on glass or other SiO_2_ materials. The first molecular monolayer is of the (CH_3_O)_3_Si(CH_2_)_3_X type (X=SH, NH_2_). In these materials NP are firmly grafted to the surface, with a very low noble metal concentration (Ag=0.36 μg/cm^2^ and Au=2.0–3.0 μg/cm^2^), this avoiding both cost and toxicity issues when the NP monolayer is used as coating for indwelling medical devices. Surface bearing spherical AgNP SAM exert only an intrinsic antimicrobial action, mainly due to the slow sustained Ag^+^ release and in part to the nanomechanical action of AgNP on the bacterial membrane.[^139^, ^140^, ^142^] An Au nanostar surface has instead no intrinsic effect, but when irradiated on the nanostars LSPR band, a thermal microbicidal effect is switched on thanks to the PTE. A reduction of 2 orders of magnitude in the surviving fraction was observed for a *Staphylococcus aureus* biofilm grown on the surface of the Au nanostars monolayer after irradiating for 30 min at 808 nm (irradiance=0.090 W/cm^2^).^[143]^ This surface is the model for switchable antibiofilm coatings for indwelling medical devices, as it allows through‐tissues laser irradiation with strong bactericidal effect, that could avoid the surgical removal of an implant in case of biofilm formation. Moreover, thermal killing is obviously full‐range effective, that is, it works on every bacterial strain, avoiding the drug‐resistance issue, that may compromise the efficiency of antibacterial surfaces based on the release of antibiotics.^[144]^ Along this approach, photothermal surfaces have been prepared with different Au nanostars featuring more than one LSPR NIR absorption, this allowing multichannel irradiation for cumulative heat generation.^[141]^ After these first examples, literature reported antimicrobial surfaces with layers of many different photothermal NP,^[145]^ such as Au nanorods (irradiation at *λ*_irr_ 808 nm),^[146]^ aggregated spherical AuNP (*λ*_irr_ 808 nm),^[147]^ graphene oxide (GO, *λ*_irr_ 1064 nm),^[148]^ reduced GO (irradiated with a solar simulator),^[149]^ polyaniline (*λ*_irr_ 808 nm),^[150]^ polydopamine (*λ*_irr_ 808 nm),^[151]^ Cs_0.33_WO_3_ NP embedded in the polydimethylsiloxane channels of a microreactor (*λ*_irr_ 808 nm),^[152]^ and MoS_2_ (*λ*_irr_ 660 nm)^[153]^

Recently, the intrinsic effect of AgNP and the on‐demand PTE have been combined in the same surface coating, by using monolayers of silver nanoplates^[154]^ and nanotriangles,^[155]^ that have large LSPR bands in the 600–1000 nm range. The intrinsic antibacterial effect is assured on the long period by Ag^+^ release. With Ag nanoplates, 0.13 μg/cm^2^ Ag are released in water in 24 h (corresponding to <3% of total Ag), allowing a logarithmic reduction >5 of the colony forming units (CFU) of planktonic *Escherichia coli* and S. *aureus* strains in contact with the surface.^[154]^ On the other hand, irradiating at 808 nm (0.26 W/cm^2^) for 20 min allows to observe a >5 and 3.8 log units CFU reduction for *E. coli* and *S. aureus*, respectively. The high photothermal microbicidal effects obtained in very short times suggested a possible synergy between the intrinsic Ag^+^ action and the local laser‐generated heat.

**Figure 4:**
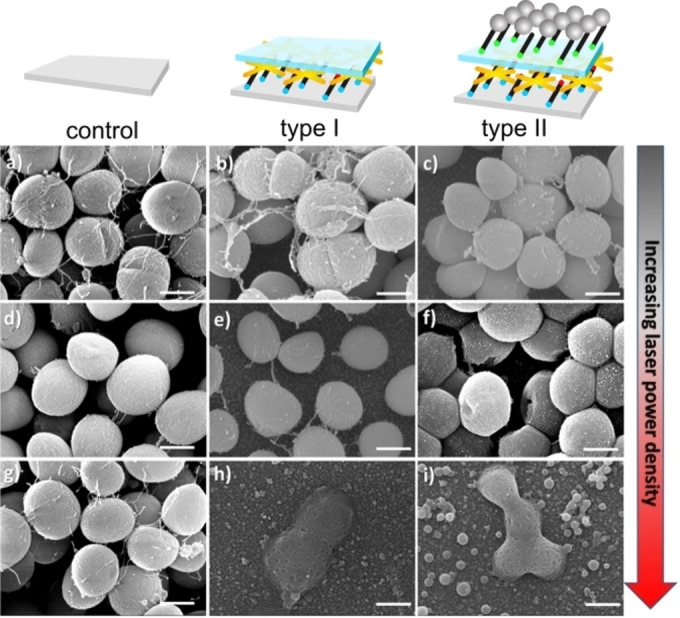
SEM images showing the effect of laser irradiation (for 10 s, using a 785 nm laser installed in a Renishaw InVia Raman Spectrometer) on *S. aureus* bacteria attached to left: plain glass; center: type I surfaces (an Au nanostars monolayer overcoated with a SiO_2_ layer); right: type II surfaces (as type I, but with a further overlayer of AgNP). Increasing power density by row: top: non‐irradiated samples; middle: samples irradiated with 5× objective lens; bottom: samples irradiated with 20× objective lens. Scale bars: 500 nm. Adapted with permission from ref. [149], published under a Creative Commons Attribution 4.0 International License (2017, Nature Publishing Group).

Such synergy was later demonstrated on a multilayer surface, with an underlying gold nanostars monolayer, separated by a 4 nm thick SiO_2_ layer from the top AgNP monolayer, type II in Figure 4. This surface and type I in Figure 4 (having an underlying Au nanostars monolayer but presenting an inert SiO_2_ surface) were tested, together with the control (plain glass slide). Pure PTE is exerted by surface type I (when irradiated at 808 nm, 0.25 W/cm^2^), while type II surface may exert either an intrinsic (with no irradiation) or an intrinsic plus PTE action (irradiating at 808 nm, 0.25 W/cm^2^). In the latter case, the sum of the logarithmic CFU reduction for short irradiation times (30 min) on planktonic cells is by far larger than the sum or intrinsic and pure photothermal actions (CFU reduction >6 log units for both *E. coli* and *S. aureus*).^[156]^ Figure 4 visually illustrates the effect on *S. aureus* bacteria adhering to the surfaces (control, type I, type II) under no irradiation and laser irradiation of increasing power. A similar approach used a multilayered coating with GO as the photothermal agent, while bearing embedded spherical AgNP further shielded by polymeric dopamine‐conjugated polysaccharide sulfate (this is needed to decrease the toxic effect played on mammalian cells by the reactive oxygen species (ROS) generated by GO irradiation).^[157]^ Beside the intrinsic antibacterial effect exerted by AgNP, the *T* increase generated by irradiating for a short time (7 min, *λ*_irr_ 808 nm) resulted in additional bacterial killing. However, it should be stressed that this was obtained using high irradiances (3 W/cm^2^).

Surface coatings combining PTE with other antibacterial agents have recently been reported. Monolayers of CuS NP on glass have large absorptions in the 800–1600 nm range, exert an antibacterial PTE when irradiated, but also slowly release the antibacterial Cu^2+^ cation, for a weak but prolonged intrinsic action.^[158]^ PTE and photodynamic therapy are obtained with nanoparticles that on irradiation are able to generate both heat and reactive oxygen species (ROS). This is the case of chitosan‐coated MoS_2_ nanosheets, that generate ROS when irradiated at 660 nm, heat when irradiated at 808 nm,^[159]^ and both ROS and heat when irradiating simultaneously with both laser sources, with efficient killing of *E. coli* and *S. aureus*. PTE is coupled with the release of an antibiotic in Ti surfaces coated with chitosan, PEG‐protected MoS_2_ nanoflakes and gentamicin. *E. coli* and *S. aureus* viability is reduced by 94 % after 120 min contact with this surface, while a >99 % viability reduction is obtained in just 5 min when switching on 808 nm irradiation, this maintaining a 50 °C local *T*. Moreover, the Δ*T* due to irradiation also induce a more than threefold increase of the gentamicin release from surface.^[160]^

### 3.4 Photothermal flexible materials: medical devices for topical use

Beside using the PTE of NP on the surface of bulk objects for a drugless and switchable antimicrobial action, some authors have proposed to use photothermal nanoparticles in solution against planktonic or plated pathogenic bacteria.^[161]^ In some cases, as against methicillin‐resistant *S. aureus* (MRSA) strains, photothermal NP were equipped with targeting antibodies, to promote adhesion to the bacterial membrane.[^162^, ^163^] However, despite successful in‐vitro experiments, the antibacterial use of the PTE of NP as colloidal solutions is limited by the high concentration needed to achieve a significant *T* increase in a large volume (e. g., in the body), due to heat dissipation by the solvent (e. g., plasma). In case of real in‐vivo use, this would lead to high risks of NP accumulation and/or to the need of high laser intensities, exceeding the allowed safe limits. A different and more promising approach has recently surfaced, by embedding photothermal NP in gels or flexible films designed for topical medications (e. g., infected wounds). An injectable silk protein hydrogel, containing spherical AuNP, was used on subcutaneous infection by MRSA in mice.^[164]^ Laser treatment can be carried out at 532 nm, even if *λ*_irr_ is out of the biotransparent window, as a very thin skin layer should be crossed. Ten minutes of irradiation reduce the CFU count to 20 % of the control, without significant skin damage. Silicone rubber films with grafted poly(acrylic acid) bind spherical AuNP and these, on 5 min laser irradiation (*λ*_irr_ 514 nm, 0.5 W/cm^2^) reduce by more than three orders of magnitude the number of CFU of *S. aureus* in contact with the film.^[165]^ Electrospinning was used to prepare poly(vinylidene)fluoride membranes containing both TiO_2_ NP and GO nanosheets. This material, irradiated at a single wavelength (980 nm, 2.5 W/cm^2^) is capable of generating both ROS (thanks to TiO_2_) and heat (thanks to GO), exerting a strong killing action against *S. aureus* and promoting wound healing on murine models.^[166]^ Although it is not a through‐tissue approach, the use of NP with NIR absorption bands is common also for these applications, as it minimizes the risk of damaging the skin region surrounding the film. Along this consideration, polyvinyl alcohol (PVA) films were prepared with embedded Au nanostars, with two large LSPR bands at *λ*_max_ 780 and >1000 nm. The CFU of *E. coli* bacteria inoculated in the film are reduced at <40 % of the control by 5 min irradiation at 1064 nm (5w/cm^2^).^[167]^ PVA films are biocompatible and have an high capability of absorbing aqueous liquids, an advantage for wound healing, as it helps to remove the wound exudate. Consequently, several preparations use this polymer as the film matrix. Prussian blue NP embedded in PVA^[168]^ lead to a ∼40 % reduction of the vitality of the Gram negative *Pneumonia aeruginosa* bacteria inoculated in the film, after 30 min irradiation (*λ*_irr_ 700 nm, 0.3 W/cm^2^). PVA films are usually obtained by casting (e. g., in a Petri dish) an aqueous solution containing PVA, a reticulant *′crosslinker′* (in most cases sodium citrate) and eventually a plasticizer (e. g., a low mw PEG). In a recent paper PVA films containing Prussian blue NP were instead sprayed on the desired surface, an approach that may increase the versatility of the use of such films. The viable fraction of *P. aeruginosa* bacteria inoculated in the sprayed films were reduced by 90 % in 15 min irradiation at 700 nm (0.3 W/cm^2^), the dead fraction of the more resistant Gram positive *S. aureus* was instead 70 % after 60 min irradiation.^[169]^ Recently, the conditions were found to obtain Au nanostars and AgNP coexisting in the same PVA film, exploiting a one pot preparation, as sketched in Figure 3D. This film exerts both an intrinsic antibacterial chemical action by Ag^+^ release, and a switchable one by irradiating at 808 nm on the Au nanostars LSPR band.^[170]^ The total content of noble metals is maintained very low (<0.15 % *w*/*w*) and in particular the potentially toxic Ag is <0.025 % *w*/*w* in this film. A reduction of 5–7 (depending on films composition) orders of magnitude is observed in the CFU of *E. coli* and *S. aureus* planktonic strains after 24 h contact with the films, whereas 80–90 % death cells are observed for film‐inoculated strains after 30 min irradiation (808 nm, 0.3 W/cm^2^).

## 4 Technological Applications of PTE Using NPs

Skin penetration, accumulation, intrinsic toxicity, toxicity due to degradation and cation/molecule release are all risks to be taken into consideration when exploiting the PTE of NP for potential in‐body or even topical use. Intrinsically safer, non‐biomedical applications of the NP PTE have been developed in parallel, at least in the most recent years. These include the use of NP to harvest sunlight, directly transforming radiation into heat or aqueous vapor, and anticounterfeit writing using photothermal nano‐inks, in which the encrypted information is not optical but thermal.

### 4.1 Photothermal nano heaters and solar steam reactors

Conventional solar thermal collectors are plates or tubes coated with materials that convert solar light into heat, increasing the temperature of an internal working fluid (water, ethylene glycol, oil). Since the first decade of the new millennium it was proposed to use instead a liquid that may be directly heated by the sun, thanks to dissolved photothermal nanoparticles.[^171^, ^172^] These type of liquids are commonly named “nanofluids”. The area flourished in the past decade, and a number of reviews appeared recently.[^173^, ^174^, ^175^] As a general requirement, NP used in this approach must absorb in the largest possible wavelength range, ideally in order to collect the entire sun spectrum.

**Figure 5:**
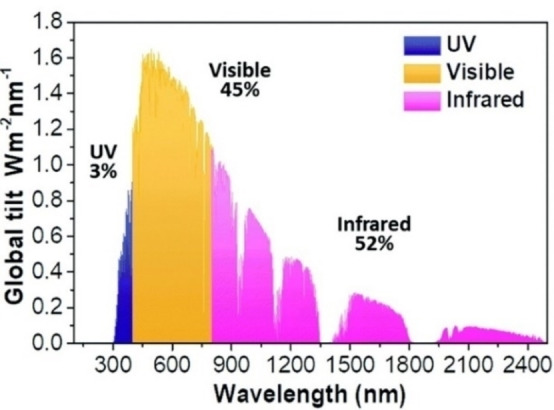
Solar spectral irradiance, adapted from ref. [181] with permission. Copyright: 2019, The Royal Society of Chemistry.

The solar spectral irradiance (Figure 5) is 3 % UV, 45 % visible and 52 % IR, ending at wavelengths as long as 2300 nm. These considerations have led to the use of NP with main absorptions in the NIR for sun‐harvesting nanofluids. To obtain an efficient and cost effective nanofluid, these NP should have low tendency to aggregate, they must be able to reach high concentrations in the chosen liquid, they must efficiently convert solar light into heat (i. e., have low/negligible luminescence) and their preparation and material must be not expensive. Despite their cost and sometimes narrow LSPR absorption bands, the use of noble metal NP for nanofluids was intensely studied. Spherical citrated‐coated AuNP (*d*=18 nm, LSPR maximum at 520 nm) in low concentrations (0.15–28 ppm) have been used for nanofluids capable of increasing by 20 % the photothermal conversion efficiency of the solvent (water).^[176]^ Larger spheroidal AuNP (*d*=49 nm) at a higher concentration (400 ppm) increase the water energy conversion efficiency by 95 %,^[177]^ whereas it is necessary to use mixtures of NIR‐absorbing Au nanorods, nanoellipsoids and nanosheets to obtain blended nanofluids capable of a wider spectral absorbance and of superior conversion efficiencies.^[178]^ However, thanks to their low cost and large‐range absorptions, carbon‐based NP are the most commonly used to obtain efficient nanofluids. Aqueous emulsion of paraffin droplets decorated with graphite nanoplatelets,^[179]^ multiwalled carbon nanotubes (mwcnt) with sodium dodecyl sulfate (SDS) in water (mwcnt/SDS 1 : 3 mass ratio),^[180]^ carbon nanohorns in water and ethylene glycol,^[181]^ graphene in ionic liquids^[182]^ have all been proven to form excellent nanofluids for the direct transformation of solar irradiation into heat. Also semiconductor NP, like those of metal oxides, have become popular to obtain nanofluids, often in the presence of high concentration of surfactants or polymers to avoid aggregation. Oleate‐coated Fe_3_O_4_ NP (*d*=10 nm) dispersed in water with high SDS concentrations (25 g/L),^[183]^ CuO NP in paraffin (with no further additives),^[184]^ CuO (*d* <40 nm) in 70 : 30 v/v water/ethylene glycol mixtures in the presence of polyvinylpyrrolydone,^[185]^ mixtures of Al_2_O_3_/Co_3_O_4_ NP in water with Triton X‐100^[186]^ have been reported as efficient nanofluids. In this area, a detailed study compared aqueous colloidal solutions of Al_2_O_3_, CuO, TiO_2_, ZnO, CeO_2_, and Fe_2_O_3_ NP, in the 0.05–1.0 % volumetric concentration range. Both the transmittance and the extinction distance of solar radiation were considered as a function of the NP material and concentration. The best features were found for CuO NP solutions, that have ∼0 transmittance for *λ*<800 nm at all concentrations. However, the extinction distance of CuO nanofluids increases from 3 mm to >1 cm in the 800–1300 nm range when the NP have low volumetric concentrations: 0.1 % was found as the ideal for real‐use nanofluids in solar collectors.^[187]^

A strictly related application of photothermal NP is the solar‐driven production of aqueous vapor to obtain clean water, that is seen as a green approach to solve the increasing water scarcity problem. Most photothermal NP used for direct heat production in nanofluids are suitable for this approach, as the primary need is identical, that is, have efficient solar absorbers converting radiation into heat. In this case, solubility and/or stability in water is also compulsory. Accordingly, NP of plasmonic metals, semiconductors and carbon‐based materials are used.^[188]^ Heat consumption through bulk heating of water, heat losses to the container and environment, convection and radiation should be minimized, and beside its absorption spectrum, the NP shape can optimize the steam generation efficiency, as in the case of hollow porous Au nanoshells, that have large LSPR absorptions from the visible up 1400 nm and when in water are able to generate vapor bubbles simultaneously from the interior and the exterior of the NP.^[189]^ Composite materials and a 3D set up may further promote efficiency: Au nanowires self‐assembled in the pores of anodic aluminum oxide (used as a template) form a highly absorbing flexible film (average absorption 91 % in the 400–2500 nm range) in which water enters in the microfunnels formed by the self‐aggregated Au nanowires, with a solar thermal conversion efficiency up to 57 %.^[190]^ Interfacial solar water evaporation is obtained with a flexible membrane of highly absorbing Carbon nanotubes interfaced with macroporous silica, with a water evaporation rate of 1.32 kg/m^2^h and solar thermal conversion efficiency of 82 %.^[191]^ Using the sun irradiation is of course considered a green approach for steam generation. In this context even greener approaches are now starting to be proposed, that use nanostructured biomaterials: 3D black carbon nanostructures were obtained by flame treatment of wood, presenting ultrahigh solar light absorption (>99 %) in the UV‐vis region and ∼97 % in the NIR (780–2500 nm), resulting in a solar thermal conversion efficiency of 72 %.^[192]^

### 4.2 Inkjet‐printed patterns of photothermal NP

In inkjet printing, nano‐inks, that is, inks made of colloidal solutions of NP, are particularly interesting, as NP can bring additional physicochemical features to the printed patterns.[^193^, ^194^, ^195^] Inks for inkjet printing need to satisfy particular ranges of surface tension and viscosity,^[195]^ these can be obtained from aqueous NP solutions by co‐solvents addition (e. g., isopropanol, ethylene glycol) and by tailoring the charge and hydrophobicity of the NP. Coatings capable of stabilizing a given NP in the chosen water/solvents mixture may thus be particularly important. Recently, nano‐inks have been described containing NP that display a PTE, like pegylated gold nanostars (PEG *M*_w_=2000)^[193]^ that were printed on glass, and Prussian blue NP (uncoated),^[196]^ printed on graphite‐based electrodes. Oleic acid‐coated CdS quantum dots dissolved in toluene were printed on glass.^[197]^ Copper sulfide NP patterns were instead obtained on paper with a post‐print treatment from inkjet printing of an aqueous Cu^II^ acetate ink.^[198]^ A method was proposed for inkjet printing layers of different types of Au NP (nanospheres and nanorods) endowed with absorption in different parts of the spectrum, Vis and NIR.^[199]^ Multiple and independent qualitative thermal readings of the patterns can be obtained by irradiating them with lasers at different wavelengths. A similar approach was used for a quantitative thermal reading by preparing inks with PEGylated Au nanostars overcoated with multiple layers of the ionic polymers poly(allylamine hydrochloride) (PAH, positive) and poly(styrene sulfonate) (PSS; negative).^[200]^ In the printed patterns, Au nanostars are kept at sufficient interparticle distance by their coatings to avoid the hybridization of their LSPR bands and maintain the same sharp absorptions observed in solution. As these bands are photothermally active, the temperature increase (Δ*T*) induced by laser irradiation follows a Δ*T* vs irradiation wavelength (*λ*_irr_) profile that has the same peak shape of the bands. Local Δ*T* of different amplitudes are thus obtained on the irradiated prints, depending on *λ*_irr_ and on the laser irradiance. Basing on this, it is possible to define a spectral response for secure reading of photothermal barcodes, that may find application in highly secure anticounterfeit tags.^[200]^

The sharpness of the LSPR bands in the printed patterns is a major requirement for obtaining significantly different Δ*T* values on changing *λ*_irr_ and thus efficiently encrypting information. A recent study on photothermal nano‐inks based on spherical AuNP (*d*=18 nm) examined in detail the coating necessary to maintain AuNP well separated in the dry prints and avoid LSPR hybridization. High molecular weight thiolated poly(ethylene glycols) (PEG‐SH, *M*_w_ 5000–20000) used as coatings are not successful, while an overlayer of PAH/PSS on a first coating layer of carboxylate‐terminated PEG‐SH allows to have sharp LSPR bands also in the prints, and to write photothermally readable secure information like a three‐wavelength photothermal barcode.^[201]^ Also applications in biology or medicine have been envisaged for 2D printed photothermal NP. The CuS NP layers obtained on latex‐coated paper from Cu^II^ acetate inks were proposed for microstructured patterns capable of thermal cell treatment, with no intrinsic toxicity, as human dermal fibroblasts can grow on top of the CuS films with no evidence of cytotoxicity.^[198]^ Inkjet printed photothermal Au nanostars on paper substrates are capable of switching the release of thiolated molecules bound to the nanostars on NIR irradiation, in a proof of concept of photothermally driven 2D drug release device.^[193]^ Inkjet printing has not been restricted to create 2D patterns, as it has been used also to prepare microcapsules containing photothermal NP for applications in cell therapy and tissue repairing. A recent work^[202]^ reports the development of a custom made inkjet printing setup to produce micron‐size droplets of chitosan containing gold nanoparticles (14 nm average size), showing a low degree of aggregation. The printed chitosan microcapsules have high adhesion ability on HeLa cells, without appreciable cytotoxicity, and they are highly resistant to degradation even at the acidic pH of gastric medium. Even though these inkjet produced micron‐size capsules have not been applied yet in specific medical treatments, they have a great potential for combined photothermal and drug delivery applications.^[203]^

## 5 Summary and Outlook

Although the photothermal ablation of tumors is the subject of the most abundant literature in the area of NPs displaying PTE, no report that stepped from cell lines or murine models to pilot clinical trials appeared until 2019, when the treatment of prostate cancer with silica‐gold nanoshells and optical‐fiber‐directed NIR laser was reported to be successful on 46 subjects.^[204]^ Potential NP toxicity and accumulation, the need of high NP concentrations, the high irradiances used in research papers and the bodily deepness of most tumors are all obstacles in the translation of photothermal through‐tissue treatments from lab benches to clinic.

The topical use of NPs displaying PTE on their surfaces (to avoid microbial contamination) or as an additional on‐demand antibacterial tool to be applied to wounds by using gels, patches, or polymeric sheets appears instead to be a safer – or at least easier – application: it minimizes the risks of NP internalization and it requires direct (i. e., non‐through‐tissue) treatments with low irradiance laser sources. Obviously, all non‐biomedical, technological applications of photothermal NPs have the same safety advantages. Moreover, the use of a relatively simple set up is implied, especially in the case of direct solar heating of nanofluids or vapor generation, that are now at a high technology‐transfer level.[^205^, ^206^]

Finally, the ability of the synthetic chemist to tailor the composition, shape dimensions and coating of NPs, and consequently their optical properties, photothermal response, and stability, allows complex, highly technological niche applications to be attained. These encompass the printing of photothermal anticounterfeit tags (e. g., for luxury goods or for encrypted writing),[^199^, ^200^, ^201^] the preparation of proteinaceous micropatterns with embedded photothermal NPs (suitable for high spatial resolution thermal stimulation of cells),^[207]^ annealing of optical‐fiber sensors bearing molecularly imprinted polymers with embedded plasmonic nanoparticles,[^208^, ^209^] or photothermally driven tuning of the pore dimensions in polysulfone membranes for water filtration.^[210]^

## Conflict of interest

The authors declare no conflict of interest.

## Biographical Information

*Piersandro Pallavicini obtained his PhD from the Scuola Normale Superiore, Pisa, Italy in 1991, and then worked at the Department of Chemistry of the University of Pavia, Italy, where he is now full professor. His main interests are in the field of supramolecular chemistry and inorganic nanochemistry applied to antimicrobial surfaces and materials, sensors, 2D printable nano‐inks. Since 2020 he has been a member of the editorial board of Nanomaterials*.

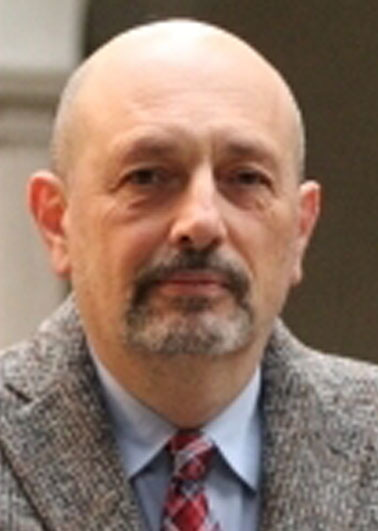

## Biographical Information

*Giuseppe Chirico received his PhD in biophysics in 1990 from the Università di Milano. He is currently Professor of Applied Physics at the University of Milano–Bicocca. His activity covers biophysics, photonics and nanotechnology, and he is currently developing photo‐ and fluorescence correlation spectroscopies and in‐vivo deep‐tissue imaging by means of nonlinear optical microscopy. Since 2021 he has been a member of the editorial board of Nanomaterials*.

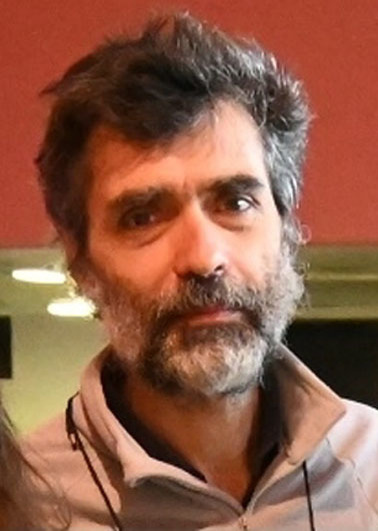

## Biographical Information

*Angelo Taglietti earned a Ph.D in chemistry in 1995 from the University of Pavia, where now he works as an associate professor. His research interests have moved from supramolecular systems to the use of transition metal complexes and nano‐objects for antibacterial purposes, and to the synthesis of nano‐devices for sensing and theranostic applications exploiting their SERS activity*.